# The views of doctors in their first year of medical practice on the lasting impact of a preparation for house officer course they undertook as final year medical students

**DOI:** 10.1186/1472-6920-10-48

**Published:** 2010-06-23

**Authors:** Catherine B Matheson, David J Matheson, John H Saunders, Claire Howarth

**Affiliations:** 1 University of Nottingham and East Midlands Healthcare Workforce Deanery, Nottingham UK; 2 East Midlands Healthcare Workforce Deanery, Nottingham, UK; 3 North West London Hospitals Deanery, London, UK

## Abstract

**Background:**

The UK General Medical Council recommends that medical students have the opportunity of shadowing the outgoing new doctor whose post they will soon undertake. At the University of Nottingham the two-week shadowing period was preceded by two weeks of lectures/seminars wherein students followed sessions on topics such as common medical/surgical emergencies, contracts, time management, surviving the first two years of clinical practice, careers advice and so on.

The present study aimed to gain a better knowledge and understanding of the lasting impact of a four-week preparation course for new Foundation Year 1 doctors [F1 s - interns]. The objectives chosen to achieve this aim were:

1/ to determine the extent to which the lecture/seminar course and shadowing period achieved their stated aim of smoothing the transition from life as a medical student to work as a new doctor;

2/ to evaluate perceptions of the importance of various forms of knowledge in easing the transition between medical student and new doctor

**Method:**

In the spring of 2007, 90 graduates from Nottingham were randomly selected and then emailed a link to a short, online survey of quantitative and qualitative questions. Of these 76 responded. Analysis of quantitative data was carried out using SPSS 16.0 and employed McNemar's test. Analysis of the qualitative data was carried out using the constant comparative method.

**Results:**

Only 31% of respondents strongly agreed or agreed that the lecture/seminar part of the course prepared them well for their first FY1 post; 14% agreed that during their first job they drew on the knowledge gained during the lecture/seminar course; 94% strongly agreed or agreed that the shadowing part of the course was more useful than the lecture/seminar part.

Experiential knowledge gained in the shadowing was the most highly valued, followed by procedural knowledge with propositional knowledge coming far behind.

**Conclusions:**

Our study shows that new doctors retrospectively value most the knowledge they are able to transfer to the workplace and value least material which seems to repeat what they had learned for their final exams.

## Background

Introduced in 2005, the Foundation Programme is a two-year generic training programme whose aim is to provide new medical graduates with the knowledge, skills and attitudes necessary for entry into specialty training - including training for general practice. Each Foundation Year is divided into three four-month posts. It is only after successfully passing the Foundation Year 1 (FY1) that a doctor is fully registered with the UK General Medical Council (GMC). The FY1 was previously known as the *pre-registration house officer*, or PRHO, year [1]. Foundation training is done under the auspices of the Foundation Schools, of which there are 25, covering the entirety of the United Kingdom. Trent Foundation School, the locus of this study, is centred on the city of Nottingham in the northern part of the English East Midlands, and contained at the time of the study two teaching hospitals [now merged into one] and numerous district general hospitals. Although teaching of medical students and junior doctors takes place in all these hospitals, UK tradition has it that only hospitals linked directly to a university [sometimes referred to as university hospitals] are termed teaching hospitals.

*Tomorrow's Doctors *[2,3], published by the GMC is a framework of recommended knowledge, skills, attitudes and behaviours expected of newly qualified medical graduates. Before and after *Tomorrow's Doctors *[2,3] there have been concerns that new graduates lacked both clinical skills and confidence and that the transition from medical student to new doctor should be more seamless [4-12]. For example, Goodfellow [4] questioned whether medical students were indeed ready to be house officers at the moment they sat their final exams and expressed concern that many were not; Jones *et al *[5] found that graduating medical students were least prepared in diagnosis and decision-making, suturing and inserting a nasogastric tube but best prepared in knowing their limitations and asking for help - a point we have confirmed elsewhere [13]; Smith and Poplett [6] found gaps in new doctors' knowledge of acute care; while Goldacre et al [7] found a lack of self-confidence among medical students as they transited into their new roles as doctors.

From 1996, the Nottingham Medical School, joined from its inception by the Trent Foundation School, have run a mandatory *Preparation for House Officer *course, consisting of a two-week [reduced to one week since 2009] series of lectures/seminars, followed by two weeks of shadowing, wherein medical students shadow the PRHO/F1 doctor whose job they are going to take over when they start work in August [14]. The course takes place just before the medical students graduate. In 2002, the Quality Assurance Agency for Higher Education commented that the course helped 'students manage the transition from senior medical student to licensed medical practitioner' [15].

In 2007, the lecture/seminar part of the *Preparation for House Officer *course consisted of sessions on various types of emergencies, fluids, sepsis, acute care, how to prescribe, how to avoid being struck off [i.e. losing one's medical licence either temporarily or permanently], medicine and the law including death certification, surviving the NHS, time management, work life balance, doctor as patient, complaints, careers in hospitals and in general practice [i.e. family medicine], how to survive the Foundation Programme and how to make the most of shadowing. Based around *Tomorrow's Doctor's *[3], the content of the lecture/seminar part of the course had evolved over the years to reflect what was felt by the course steering group to be essential revision for the new doctors. The timetable of lecture/seminar part is shown in Additional File 1.

The shadowing fulfilled the GMC recommendation that:

Students should have opportunities to shadow the PRHO in the post that they will take up when they graduate. Such attachments allow students to become familiar with the facilities available, the working environment and to get to know their colleagues [3].

The University set general requirements on the shadowing as detailed in Additional File 2 to which the hospitals added their own sessions, including lunchtime teaching, as shown in Table 1.

**Table 1:** Typical shadowing timetable

Week 1			
Mon 18.06	10.00 a.m.	Welcome	Director of Postgraduate Medical Education; Foundation Year 1 Tutor; Medical Education Manager; HR Administrator; Accommodation Manager, LHA; Senior Occupational Health Nurse
	
	10.45 am	Meet own teams	
	
	12.15 - 12.30 pm	ID badge photographs/Car permits	XXX, Fire & Security Officer/Excel Parking
	
	1.00 - 2 pm (lunch provided from12.30 pm)	Record Keeping	XXX, Director of Postgraduate Medical Education; XXX, F1 Doctor

Tues 19.06	09.30 - 12.30 pm **(GROUP A)**	ILS Recertification Course	Resuscitation Team: Venue: Lecture Theatre, rooms 6/7&8

Wed 20.06	1.00 - 2 pm	ACAT Scoring	XXX
	
	10.30 - 11.30 am	Path Lab procedures **(GROUP A)**	Senior Scientists Pathology Services **Venue: Path Labs**

	1.00 - 2 pm (lunch provided from12.30 pm)	Pharmacy Matters	XXX, Lead Pharmacist
	
	2.00 pm - 5.00 pm	ALERT Refresher Course, (rooms 3&4/6)	XXX

Thurs 21.06	10.00 - 10.45 am	Blood Gas Analyser **(GROUP A)**	XXX: **Venue: AMU**
	
	1.00 - 2 pm (lunch provided from12.30 pm)	Antibiotics & Hospital Acquired Infections	XXX, Consultant Microbiologist

Fri 22.06	1.00 - 2 pm (lunch provided from12.30 pm)	Coping with relatives, Death Certificates	XXX, Consultant, General Medicine; Reverend XXX, Hospital Chaplain

**Week 2**			

Mon 25.06	1.00 - 2 pm (lunch provided from12.30 pm)	Introduction to General Medicine	XXX, Service Director, Medicine;
	
	2.00 - 3.00 pm	Radiology	XXX

Tues 26.06	1.00 - 2 pm (lunch provided from12.30 pm)	IT Training	XXX, IT Trainer: Venue: Lecture Theatre, Education Centre

Wed 27.06	1.00 - 2 pm (lunch provided from12.30 pm)	Introduction to General Surgery	Service Director, General Surgery; Rota Co-ordinator, Surgery

Thurs 28.06	1.00 - 2 pm (lunch provided from12.30 pm)	Employment Contracts & HR Issues	XXX, HR Advisor; XXX, HR Administrator

Fri 29.06	1.00 - 2 pm (lunch provided from12.30 pm)	Foundation Programme Assessment/Portfolio	Medical Education Team

All non-timetabled time to be spent on Wards

Like Nottingham, and in keeping with *Tomorrow's Doctors *[3], many medical schools and/or Foundation Schools have set up short courses that include a period of shadowing with the aim of smoothing the transition from medical student to new doctor. There is an on-going debate on the value and usefulness of short transitional courses in helping new doctors prepare for practice. Whitehouse et al. [16] and Jones et al. [17] evaluated seven-week shadowing programmes undertaken at the end of the final undergraduate year. They reported student satisfaction, better preparedness and increased levels of confidence. Evans et al. [8] and Berridge et al. [11] evaluated an extended five-day induction and a two-week programme, respectively, that took place immediately before the Pre-registration House Officer starting date. Both programmes included clinical skills training and shadowing the outgoing house officer to facilitate the transition between medical student and new doctor. Evans et al found that 'newly qualified doctors do not feel prepared for PRHO duties and objectively are not competent in basic clinical skills' [9] which concurs with our own findings from another study [13] while Berridge et al found that the programme they reported upon was useful in supporting the transition from medical student to practising doctor but would have benefited from the student being given more responsibility during the shadowing period [11].

Jones et al. [17] collected qualitative data (23 interviews) three months into the PRHO year. Berridge et al. [11] collected both qualitative (12 focus groups and 86 participants) and quantitative data at the start and end of the course and one month into the PRHO year (50, 34, 35 questionnaires respectively). As we wanted to examine the lasting impact, we collected both quantitative and qualitative data 8-10 months into the Foundation Year 1 [FY1] in order to gain a better knowledge and understanding of the lasting impact of a four-week preparation course for new Foundation Year 1 doctors.

The objectives chosen to achieve this aim were:

1. to determine the extent to which the four-week course achieved its stated aim of increasing confidence and consolidating attitudes, skills and knowledge required for safe and effective practice as an F1;

2. to evaluate perceptions of the importance of various forms of knowledge in easing the transition between medical student and new doctor.

## Methods

The sample for the present study was randomly drawn to represent half the F1 s who had graduated from the five year course from Nottingham Medical School and who worked in the Trent Foundation School. One half of the sample worked in the two University hospitals and the other half in the general district hospitals associated with the Trent Foundation School. They were contacted by email during their final four-month post of FY1 and invited to take part in an anonymous online survey (the questionnaire used in the survey is in Additional File 3). As the survey was an evaluation of a University course and we were approaching potential respondents as University alumni, no ethical approval was required and exemption was granted by the University of Nottingham Research and Innovation Committee. The 90 potential respondents were emailed three times during their third attachment (April, May and June), by the end of which time 76 (84%) had responded to the questionnaire. As the questionnaire was anonymous, no data was collected on sex, ethnicity, age, religious beliefs and sexual orientation. The email contained a link to the survey which was hosted on SurveyMonkey.

The questions aimed at gathering both quantitative and qualitative data in order to evaluate how well the four-week course prepared final year medical students for FY1 and did so by focussing on perceptions of overall preparedness, what was most and least useful about both the lecture/seminar part and the shadowing part of the course, whether there was anything in the lecture/seminar part of the course that seemed unimportant at the time but was perceived to be important for a new F1 as well as suggestions about how to improve the four-week course in future. For the sake of clarity, the first part of the course is referred to hereafter as the lecture/seminar part of the course. However in the survey, and in the University, it is termed the taught part, even though teaching occurs in the shadowing as we see in Table 1.

The quantitative data was collected through 11 questions using a mixture of Likert scale (strongly agree = 5; agree = 4; uncertain = 3; disagree = 2; strongly disagree = 1) and respondents selecting from several itemised responses. In several instances, similar questions were phrased negatively and positively to reduce bias due to acquiescence effect [18] and maximise the internal consistency, validity and reliability of the data [19]. The quantitative data was analysed using SPSS 16.0. The qualitative data was collected through eight open-ended questions. The qualitative data was analysed using the constant comparative method [20].

## Results

### Overall views of how well the Preparation for House Officer course prepared medical students for FY1

As shown in Table 2, overall the respondents felt that the lecture/seminar course did not prepare them well for working as F1 s. The shadowing was perceived to be more useful than the lecture/seminar part.

**Table 2:** How well the *Preparation for House Officer *course prepared medical students for FY1

Questions	strongly disagree 1	disagree 2	don't know 3	agree 4	strongly agree 5	mean	SD
**The taught part of the course prepared me well for my first FY1 job [n = 74]**	11% [8]	45% [33]	14% [10]	28% [21]	3% [2]	2.7	1.09
**The taught part of the course did not prepare me well for working as an F1 [n = 72]**	1% [1]	18% [14]	8% [6]	54% [40]	15% [11]	3.6	1.01
**During my first job I drew on the knowledge gained during the taught course [n = 74]**	18% [13]	45% [33]	23% [17]	14% [10]	1% [1]	2.4	.97
**The taught part of the course gave me better preparation than the shadowing part [n = 74]**	59% [44]	34% [25]	5% [4]	1% [1]	0%[ 0]	1.5	.67
**The shadowing part of the course was more useful than the taught part [n = 74]**	1% [1]	1% [1]	3% [2]	39% [29]	55% [41]	4.5	.74
**A lot of what we did in the taught part should have been done during shadowing [n = 74]**	3% [2]	12% [9]	22% [16]	47% [35]	16% [12]	3.6	.99

Only 31% strongly agreed or agreed that the lecture/seminar part of the course prepared them well for their first FY1 post while 14% did not know. In contrast, 69% strongly agreed or agreed that the lecture/seminar part of the course did not prepare them well for working as an F1 while only 8% did not know. Strongly agreed/agreed were combined as one category and strongly disagreed/disagreed as the other while 'don't knows' were eliminated from the analysis - n to 61.

We see from Table 2 that 23 people agreed or strongly agreed with the proposition (which we will call proposition p1) that 'The taught part of my course prepared me well for my first FY1 job' and that 41 disagreed or strongly disagreed. We also see from Table 2 that 51 people agreed or strongly agreed (we will call this proposition p2) that 'The taught part of my course did not prepare me well for working as an F1' and that 15 disagreed or strongly disagreed. However SPSS discounts those who only responded to one of the two propositions, hence the totals below are lower than in Table 2.

In order to use McNemar's test, we need to reverse the scoring of proposition 2 and cross-tabulate as shown in Table 3.

**Table 3:** p1 versus p2

	p2 disagree	p2 agree	total
**p1 agree**	36	4	40

**p1 disagree**	11	10	21

**Total**	47	14	61

With this reversal McNemar's test gave a p value of .121, showing there is no statistically significant difference between responses to p1 and reversed responses to p2.

Only 14% agreed that during their first job they drew on the knowledge gained during the lecture/seminar course and nearly a quarter (23%) said that they did not know.

A total of 93% strongly disagreed or disagreed that the lecture/seminar part of the course gave better preparation than the shadowing part (proposition p3 in the table below) while 94% strongly agreed or agreed that the shadowing part of the course was more useful than the taught part (proposition p4 in the table below). Strongly agreed/agreed were combined as one category and strongly disagreed/disagreed as the other while 'don't knows' were eliminated from the analysis - n to 70. Applying McNemar's test as above to these propositions, we have as shown in Table 4.

**Table 4:** p3 versus p4

	p4 disagree	p4 agree	total
**p3 agree**	0	1	1

**p3 disagree**	2	67	69

**total**	2	68	70

There is no statistically significant difference between responses to p3 and reversed responses to p4 (p = 1.000).

A total of 63% strongly agreed or agreed that a lot of what they did in the lecture/seminar part should have been done during shadowing while 22% did not know and 15% disagreed. In addition, 73% thought the lecture/seminar course should be reduced to one week and 27% that it should remain a two-week course.

### Shadowing

The overwhelming majority of the respondents felt that shadowing part of the course had been more useful than the taught part in preparing them for practice.

**Q: ***Which part/s of the shadowing were most relevant to your starting work as a Foundation doctor? Can you explain your response? *The most relevant parts of shadowing for starting work as an F1 were given by 50 respondents as direct experience of actually doing the job of an F1 and becoming familiar with how the ward works and its weekly and daily routines. More specifically shadowing on-call and nights was described as most relevant and useful by 12 respondents followed by how to use bloods/XRs requests and ward rounds, each highlighted by 8 respondents.

However the key thing I found was beginning to make the transition between observing and acting. As a medical student you don't realise that you spend much of your time observing, not being part of what is going on. So, you naturally hang back, you don't want to impede the working of the team etc. However, as a doctor, you are the team. Thus I found it very helpful to begin to start thinking and doing things for myself, not watching others doing them

**Q: ***What should have been left out of the shadowing? Can you explain your response? *A total of 64 respondents were unable to say what should have been left out of shadowing or what was the least useful part of shadowing. They either did not know or indicated that nothing was irrelevant. However, 4 respondents highlighted that the compulsory lunchtime and/or afternoon teaching sessions for all F1 s were the least useful and that they should have spent their two weeks of shadowing entirely on the wards.

**Q: ***What else should have been included in the shadowing? Can you explain your response? *Ranked first among suggestions for improving shadowing was more time actually shadowing on the wards, offered by 8 respondents, although only 3 respondents thought shadowing should be extended to a third week. Typical comments were

*Way too much of the shadowing time was taken up by having to go to taught things. We never had a full day shadowing so you never got an idea of what a whole day was like and often missed the one thing you really needed to see*.

*Often times I noticed that current HOs *[house officers] *were letting incoming HOs home early. I think that if we're shadowing we should be doing the full day with our outgoing F1 including days when they needed to stay late - that's an important learning experience as well*.

More time actually spent on the wards was closely followed by compulsory night, week-end, on-call shadowing and formal IT sessions on how to use computers, each suggested by 6 respondents.

### Lecture/seminar course

Just under a third of the sample strongly agreed or agreed that the lecture/seminar part of the course had prepared them well for practice.

**Q: ***Which part/s of the taught course do you think were most relevant to your starting work as a Foundation doctor? Can you explain your response? *A total of 18 respondents did not comment and 10 said they did not know when asked about the most relevant part/s of lecture/seminar course. Ranked first as most relevant for starting as an F1 was the lecture about death certificates (19 respondents) closely followed by the sessions on prescribing/writing drug cards (15 respondents) and acute care (14 respondents). Typical comments were:

*Filling out death certificates - directly relevant to the job and something we hadn't previously covered. Prescribing lecture - again, directly relevant*.

All the parts that were new to us (death certification is the only one that really springs to mind) were quite obviously going to be relevant

*How to prescribe, although at the time this took a long time to teach us little, I do still remember a few important points learnt in that session*.

*Reminders and additional information regarding how to manage acutely unwell patients. In one's first month in particular, that's the kind of thing you fear the most, and I think is most crucial. Initial management of acutely unwell patients is absolutely key*.

**Q: ***What should have been left out of the taught course? Can you explain your response? *A total of 42 respondents did not comment or said they could not remember or did not know. Ranked first as to be left out of the lecture/seminar course was recapping medical lectures (19 respondents). A typical comment was:

*We do not need to go over (for example) the different types of jaundice again... just what to do when we have a jaundiced patient*.

**Q: ***Were there any part/s of the taught course that seemed unimportant at the time but which you now think are important for a new Foundation doctor? Can you explain your response? *A total of 37 respondents made no comments or said they could not remember or did not know while 20 respondents indicated that no part of the lecture/seminar course seemed not important at the time but was important retrospectively. Ranked first as important retrospectively was time management (7 respondents) followed by death certification (2 respondents). Typical comments were:

There was nothing in the taught course that hadn't already been covered - time would have been better spent on the wards

*All the parts that were new to us (death certification is the only one that really springs to mind) were quite obviously going to be relevant*.

*Time management seemed a bit like being taught to suck eggs, but actually had some useful parts I can remember*.

*The ones that seemed unimportant at the time still seem unimportant (time management, deanery talk (no info given), setting up your email, elective debrief)*.

**Q: ***What else should have been included in the taught course? Can you explain your response? *Suggestions for improving the taught course emphasised the need for practical information like how to prescribe, how to manage patient's medications, how to work with the pharmacists, how to fill in an insulin chart or a fluid chart, how a sliding scale works, and how to respond to on-call emergencies. Another point offered was that more sessions should have been taught by FY1 doctors. Many respondents said that sessions from the taught course should not be repeated during shadowing. How to improve the taught course was neatly encapsulated by one respondent:

*It should be much more targeted to the practical issues of starting work. We had all proved that we knew the medical theory by passing our finals, what you need before starting work is advice on how to apply that knowledge practically, for instance the most common calls at night could be covered in a brief concise way, instead of "GI emergencies" it should be "called to see a patient with abdominal pain, important things to do and not to miss" and then go through systematically how you should approach this e.g. assess the patient, read the notes, look at the obs *[i.e. observations] *chart, look at the drug card, important investigations to do before getting senior help, this is all quite basic but would really help to increase confidence before starting work and much better to get F1 s to think about it during the preparation course than on their first night shift*.

## Discussion

Overall the four-week course did achieve its stated aim of increasing confidence and consolidating attitudes, skills and knowledge required for safe and effective practice as an F1. The two-week opportunity to shadow the outgoing F1 was deemed by 93% of respondents 8-10 months after it had taken place to be the most useful and effective part of the *Preparation for House Officer *course. Hence, acquiring experiential knowledge [21,22] was deemed the most useful type of learning offered by the four-week transitional *Preparation for House Officer *course.

Nearly two-thirds of the respondents strongly agreed or agreed that a lot of what they did in the lecture/seminar part should have been done during shadowing. It is difficult to determine the extent to which this was the consequence of several sessions from the taught course having been repeated in shadowing (examples given included death certification, pharmacy, child protection and workplace-based assessment tools). Although 73% of the sample respondents said the lecture/seminar course should be shortened to one week, only 4% wanted the shadowing to be extended to three weeks. Nonetheless, many respondents emphasised that *too much of the shadowing time was taken up by having to go to taught things *and that they would have benefited from uninterrupted experiential learning during shadowing, rather than having to leave the ward to attend teaching sessions at lunch time and on some afternoons. There is a clearly a question-mark raised in respondents' minds as to what the hospitals felt the shadowing was for and this indicates a need for clarity of expectations and a delineation between the teaching in the lecture/seminar part and in the shadowing so that when there is repetition of topics, it is made clear why such apparent repetition is occurring.

**Figure 1:**
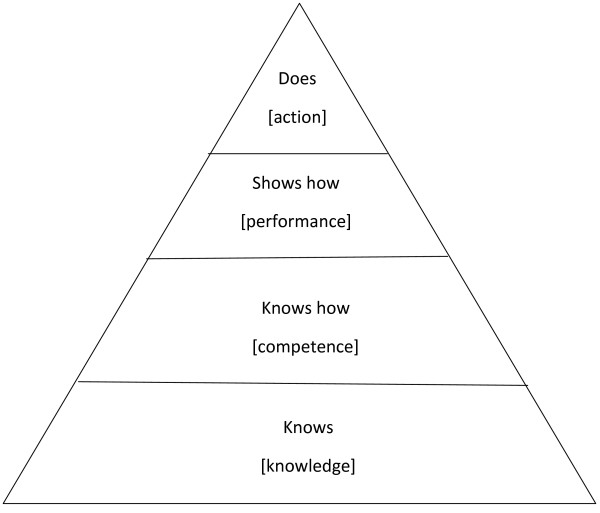
**Miller's *Pyramid ***[23].

Many respondents stated that a lot of the knowledge-based lectures during the two-week lecture/seminar course had seemed important at the time, but had not helped them *knowing what to do with a sick patient*. The majority of respondents pointed out that the two-week taught course should have focussed on *how you would manage a patient with chest pain when isolated on the wards*, rather than *the causes and physiology of a myocardial infarction*. The least relevant part of the taught course was thus identified as *the repetition of facts learnt for finals*, in other words, the recapping of already known *knowing that *or propositional knowledge. Thus, the demand was to work at least at the second level of Miller's *Pyramid *[23] shown in Figure 1.

In our study, experiential knowledge [21,22] was ranked as most relevant and propositional knowledge [23,24] as least relevant. In this respect, the respondents seem to ascribe the highest value to the more subtle knowledge of acculturation to the role of F1 doctor. In other words, they value most highly those aspects of learning that might be seen as getting them to think and behave like doctors and to internalise the cultural norms and behaviours associated with the profession [25].

While Ryle [26] underlined the importance of knowing how to do something or procedural knowledge, which cannot be reduced to any set of facts and can only be demonstrated through practice, Eraut [27] pointed out that practical or applied knowledge involves mainly procedural but also propositional knowledge and that the boundary between them is blurred. Practical knowledge came second in order of relevance. Unsurprisingly the respondents recognise that 'there is more to the practice of medicine than knowing ... and graduates must also *know how *to use the knowledge they have accumulated' [emphasis in original] [23]. Indeed it is clear from their responses that the respondents see themselves as already *knowing that *but felt themselves in need of *know-how *for immediate patient management in order to develop competence and thence *show how *[or performance, in the words of Miller] [23]. Overall, respondents highlighted the key importance of this type of knowledge at a time of transition between medical student and new doctor. Respondents suggested that practical knowledge should emphasise how to

• fill in death certificates

• prescribe

• work with the pharmacists

• manage a patient's medications

• fill in an insulin chart

• fill in a fluid chart

• work a sliding scale

• respond to on-call emergencies (using specific situations such as patients who fall, have a low blood pressure, high potassium, spike in temperature and/or severe pain)

• prioritise urgent versus immediate needs

• balance your workload

• know what important investigations to do before getting senior help

Obviously, there are some things that can only be learnt through practice and experience. However, in preparing final year medical students for their future role as new doctors, we have to encourage and facilitate future experiential learning through practical knowledge [27]. Respondents emphasised the usefulness and positive long-term impact of teaching sessions that involved practical knowledge and hence both procedural and propositional knowledge such as those on prescribing and death certification, and, to a lesser extent, acute care and time management.

While the case for experiential knowledge at this transitional stage and beyond does not have to be argued, an argument can be made for the importance of propositional knowledge when directly related to procedural knowledge and when directly relevant to the professional role of new doctor.

## Conclusions

A limitation of the study is that it was limited to one medical school and one Foundation School and that the sample, although representative, was relatively small. Another possible limitation is the problem of recall [28] or, perhaps the respondents found it impossible to prioritise the elements and thus opted for 'don't know' or left the free-text blank. In the quantitative part of the study, respondents who indicated 'don't know' ranged from 5% to 23% depending on the question. The percentage was far higher in the free-text questions as 55% and 49% did not respond or said they did not know when asked about what in the taught course had been most and least useful, respectively, for starting work as a new doctor.

**Figure 2:**
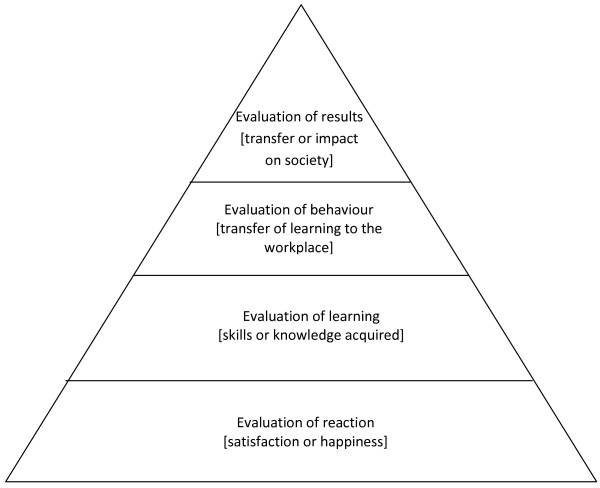
**Kirkpatrick's *Levels of Evaluation ***[29].

Although recall is less of an issue with evaluation at the end of a course, such student satisfaction surveys relate only to the students' immediate impressions of the course concerned and are hence aimed at the lowest level of Kirkpatrick's *Levels of Evaluation *[29] - shown as Figure 2 - as they cannot determine lasting impact. Our study invited respondents to consider the knowledge or skills acquired [Kirkpatrick's Level 2] in the *Preparation for House Officer Course *and the transfer of these to the workplace [Level 3] and showed that in the respondents' perceptions the most useful content was the part of the course which relates to the GMC recommendations on shadowing [3]. In addition, respondents were able to state those aspects of the lecture/seminar course that they took into the workplace as well as stating what they students themselves feel would have benefited them most in the lecture/seminar part of the course and which should be taken into account in future revisions of the course. In this way, the study provides new insights into the value and usefulness of transitional courses to prepare new doctors for practice.

Final year medical students do benefit from being exposed to experiential [21-23] as well as procedural [23,26] and even propositional knowledge [23,24] in order to acquire the necessary competencies to practise. However, more research is needed to determine what types of learning are more effective in easing the transition from final year medical student to first year new doctor and thus meeting the learning outcomes set out in *Tomorrow's Doctors *[3].

## Competing interests

The authors declare that they have no competing interests.

## Authors' contributions

The idea for the study originated with JS and CH; DM created the questionnaire and analysed the quantitative data; CM analysed the qualitative data. CM and DM wrote the article and the others commented on drafts. All the authors have read and approved the final manuscript

## Pre-publication history

The pre-publication history for this paper can be accessed here:

http://www.biomedcentral.com/1472-6920/10/48/prepub

## Supplementary Material

Additional File 1 **The timetable for the lecture/seminar part of the course**. Timetable of taught sessions for each of the two weeks of the course, with names of contributors. Click here for file

Additional File 2 **Shadowing course certificate of satisfactory completion and shadowing checklist**. Details of aims of shadowing, sign-off, checklist of activities to be carried out in the course of shadowing. Click here for file

Additional File 3 **Questionnaire used for survey of respondents**. pdf of questionnaire used in survey. Click here for file
