# Supplementary material for: The views of doctors in their first year of medical practice on the lasting impact of a preparation for house officer course they undertook as final year medical students
Source: BMC Med Educ. 2010 Jun 23;10:48. doi: 10.1186/1472-6920-10-48 (PMC2909973; doi:10.1186/1472-6920-10-48)
Supplement: Additional File 1 — The timetable for the lecture/seminar part of the course. Timetable of taught sessions for each of the two weeks of the course, with names of contributors. [file 1472-6920-10-48-S1.PDF]

PREPARATION COURSE FOR NEW DOCTORS – TIMETABLE JUNE 2007 – WEEK 1

“Exploding the Myths about August – Looking after your patients and being an effective Foundation Trainee”

VENUE: QMC LT1 initially unless otherwise stated

TIME	MONDAY 4 JUNE	TUESDAY 5 JUNE	WEDNESDAY 6 JUNE	THURSDAY 7 JUNE	FRIDAY 8 JUNE
9.00am – 10.30am	• **Introduction**<br>• **How to pass**<br>(Dr A)	**Child Protection**<br>Dr B	**Surgical Emergencies 1**<br>(Mr C and Colleagues)	• **Psychiatry** (Dr D)<br>• **Infections** (Dr E)	**Medicine and Law**<br>(Dr F & Colleagues)
10.30am – 11am	Break	Break	Break	Break	Break
11.00am – 12.30pm	**Elective Presentations**	11.00 am<br>**Neurological Emergencies**<br>(Dr G)<br>11.45 am<br>**Fluid Balance**<br>(Dr H)	**Surgical Emergencies 2**	11.00 am<br>**Respiratory**<br>(Dr I)<br>11.45am<br>**Gastroenterology**<br>(Dr J)	**Medicine and Law Continued**
12.30pm – 1.25pm	Lunch	Lunch	Lunch	Lunch *(Medical Sickness)*	Lunch
1.30pm – 2.30pm	**CVS Emergencies**<br>(Dr K)	**Sepsis**<br>(Dr L)	**How to Prescribe 1**<br>(Dr M + Colleagues)<br>(LT1 check noticeboard)	Therapeutics<br>**Pain Relief**<br>(DrN)	**OFF**
2.30pm – 3pm	Break	Break	Break	Break	
3.00pm – 4.00pm	**Interface with the Community and Community Services**<br>(Dr O)	**Acute Care and Resuscitation Skills**<br>(Dr P)	**How to Prescribe 2**	**GMC “How not to get struck off”**<br>(Dr Q)	**OFF**
4.00pm – 5.00pm	Therapeutics<br>**Terminal Care**<br>(Dr R)	**Personal Study**	**Personal Study**	**Personal study**	**OFF**
Co-ordinator	Dr K	Dr K	Dr K	Dr K	Dr K
F1 Facilitator	Dr S	Dr S	Dr S	Dr T	Dr T

Contact Tel: *ABD, Faculty Office,* *Dr K, QMC 123000*
CDF, Course Administration, Univ 123000 *Dr A, QMC 123000*

5th Yr Student Reps: *Mr U*
Mr V

Registers will be circulated each morning and afternoon.

PREPARATION COURSE FOR NEW DOCTORS – TIMETABLE JUNE 2007 – WEEK 2

“Exploding the Myths about a Successful Career – Looking after yourself and making the most of your Foundation Programme opportunities”

VENUE: QMC LT1 initially unless otherwise stated

TIME	MONDAY 11 JUNE	TUESDAY 12 JUNE	WEDNESDAY 13 JUNE	THURSDAY 14 JUNE	FRIDAY 15 JUNE
9.00am – 10.30am	**Making the most of Foundation Training** (Mr AA)	**Complaints and how to prevent them** (Dr BB)	**CAREERS DAY**<br><br>**Hospital Careers** (Trent Deanery Colleagues)	**Making the most of shadowing/post F2 selection into specialties** (DR CC and Ms DD)	**OFF**
10.30am– 11.00am	Break	Break	Break	Break	
11.00am – 12.30pm	**Personal Protection Training “Hands Off”** (Mr EE)	11.00am **EWTD & Contracts Clinic** (Ms FF)<br><br>**Deanery Slot**	**Hospital Careers Continued**<br><br>12.00noon **Session for Overseas Students Only** (Mr GG)	**Individual Sign off and Feedback** (Dr A et al)	**OFF**
12.30pm – 1.25pm	Lunch *(MDU stand)*	Lunch	Lunch *(MPS stand)*	Lunch	
1.30pm – 2.30pm	**Surviving the NHS** (Dr HH)	LT4 (1/2 year alternating)<br><br>**“Doctor as Patient”** (Dr II + Colleagues	LT1 and small groups 1.30pm – 3.30 pm **Career Forum for General Practice** (Dr II & VTS)	**COLLECT COPY OF CERTIFICATE**	**OFF**
2.30pm – 3.00pm	Break	2.45pm – 3.15pm Break	Break	Break	
3.00pm – 4.00pm	**Time Management** (Dr JJ and Dr KK)	LT4 (1/2 year alternating) 3.15pm to 4.30pm **“Doctor as Patient”** (Dr II + Colleagues)	3.30pm **Strawberries and Bucks Fizz** (sponsored by MPS)	3.00pm – 3.30pm ***OPTIONAL SESSION***<br><br>***MLP – Financial Planning Workshop***	**OFF**
4.00pm – 5.00pm	**Personal Study** (see mentor by arrangement)	**Personal Study**	**Personal Study** Complete Certificates	**OFF**	**OFF**
Co-ordinator	Dr II	Dr II	Dr II	Dr II	
F1 Facilitator	Dr LL	Dr MM	Dr MM	Dr MM	

CDF, Course Administration, Univ 123000 Dr A, QMC 123000 Mr V
