# Supplementary material for: The views of doctors in their first year of medical practice on the lasting impact of a preparation for house officer course they undertook as final year medical students
Source: BMC Med Educ. 2010 Jun 23;10:48. doi: 10.1186/1472-6920-10-48 (PMC2909973; doi:10.1186/1472-6920-10-48)
Supplement: Additional File 2 — Shadowing course certificate of satisfactory completion and shadowing checklist. Details of aims of shadowing, sign-off, checklist of activities to be carried out in the course of shadowing. [file 1472-6920-10-48-S2.PDF]

PREPARATION COURSE FOR NEW DOCTORS 2007

Shadowing Course 18 June to 30 June — Certificate of Satisfactory Completion

This is to confirm that ____________________ (Name of student)

Has/has not satisfactorily completed the above course (see details below and over)

Important *Instructions*

Completion of the full course is the last mandatory part of Medical School. This certificate, along with the Seminar certificate, will contribute towards your Educational Portfolio as an F1 doctor, and should be discussed with your Educational Supervisor when in post. Please keep both certificates safely in your Educational Portfolio.

The Medical Course Office requires a complete copy of each certificate.

The deadlines for return of certificates are:

- Seminar Course Certificate **FRIDAY 15 JUNE 2009**
- Shadowing Course Certificate **WEDNESDAY 1 JULY 2009**

A copy of this certificate, along with the two feedback questionnaires must be returned to the Medical Course Office.

Aims *of Shadowing*

1. To increase confidence for the F1 year by obtaining hands-on experience of routine ward work and acute takes
2. To obtain practical experience of ordering tests, obtaining results, dealing with death, referring patients
3. To write up prescriptions* and TTO discharge letters under supervision (*all prescriptions need to be signed by a doctor)
4. To start to get to know the nursing staff and ward personnel you will subsequently be working with

You should consider being assessed and signed off in one of the Foundation Core Competencies (e.g. ECG) during the shadowing period. Congratulations and best wishes for your new responsibilities.

If you have any queries regarding this certificate please contact:
at the Medical Course Office
B Floor, Medical School on 0115

To be completed by *Consultant*

Attended Shadowing Course	☐ (please tick)
Passed Competency Based Assessments	☐
Completed Shadowing checklist (see over)	☐
Considered Personal Objectives and areas for Further Development (see below)	☐
Consider GMC Practical Procedure checklist (see over)	☐

Signed by Consultant: ____________________ Consultant Name and Address:

Signed by Student: ____________________

Date: ____________________

Comments (if necessary):

To be completed by *Student*

PERSONAL OBJECTIVES for shadowing course and how met:

AREAS FOR FURTHER DEVELOPMENT AND EDUCATIONAL NEEDS AS F1 TRAINEE (complete now and discuss with Educational Supervisor when in post)

If necessary, please continue on a separate sheet

PREPARATION COURSE FOR NEW DOCTORS 2007

Shadowing Checklist

AND RECORD SHEET [Complete at least 17 of 22 – please tick]

1. Shadow House Officer, attend ward rounds and other team activities* ☐
2. Spend a day with Ward Sister/Senior Nurse* ☐
   -to gain insight into the working of the ward; team work; firm dynamics
3. Spend half a day in CCU or theatres, as appropriate ☐
4. Go on acute take, including one at night and discuss arrangements with F1* ☐
   Find out information about practical aspects of the job
5. Write prescriptions under supervision including IV fluid administration* ☐
   (all prescriptions need to be signed by a doctor)
6. Ordering tests:
   -what tests are available/how long tests take to perform ☐
   -when are results available/reference values ☐
   -imaging: what tests/procedures available / when to request, ie US/MRI/CT ☐
7. Useful contacts; how to contact various people / telephone numbers ☐
8. Referrals to other members of the medical / nursing / rehabilitation team ☐
9. Patient discharge (including TTOs and informative interim discharge letter)* ☐
10. Dealing with the death of a patient, including role of coroner ☐

Discuss with staff:

11. The local arrangements for theatres, procedures, DNR ☐
12. IT facilities, good notekeeping, library, reference facilities ☐
13. Educational opportunities, how to use your PRHO Portfolio ☐
14. Resuscitation Course* ☐
15. Revise GMC practical procedure checklist (see opposite) ☐
16. Complete "My Medical Career Pathway" Portfolio Sheet including discussion* ☐
17. Undertake personal objectives* ☐
18. Complete feedback questionnaire (and return to Faculty Office)* ☐
19. Arrange appraisal with consultant* ☐

*** Indicates compulsory task**

**Student Name**	CAPS
**Consultant of first F1 Job**	CAPS
**Hospital and Location of first F1 job**	CAPS

GMC PRACTICAL PROCEDURE CHECKLIST

Practical skills frequently required by F1 Trainees. Any of the following skills may be required from the start of the F1 (Pre-Registration) year; you are strongly advised to complete the items in bold italics – (tick off when done)

Practical Skills

1. Be aware of guidance on consent (see: www.doh.gov.uk/consent)
2. Calculate drug dosage accurately
3. ***Write a prescription***
4. Procedures involving veins;
   - ***Venepuncture***
   - ***Insert cannula into peripheral vein***
   - Give intravenous injections
   - Mix and inject drugs into intravenous bag
   - Use a pump to give drug treatment
5. Give intramuscular and subcutaneous injections
6. Arterial blood sampling
7. Suturing
8. ***Perform an ECG;***
   - Conduct an exercise ECG
9. ***Basic cardiopulmonary resuscitation***
10. Perform basic respiratory function tests
11. ***Administer oxygen therapy safely***
12. Correct use of a nebuliser
13. Gastrointestinal;
    - Insert nasogastric tube
    - Proctoscopy
14. Bladder catheterisation
15. Lumbar puncture (for diagnostic purposes)
    (not expected to be undertaken as a student)
16. ***Control of haemorrhage***

NB "The New Doctor" publication lists more advanced procedures expected to be carried out by the end of the F1 year
