# Supplementary material for: The views of doctors in their first year of medical practice on the lasting impact of a preparation for house officer course they undertook as final year medical students
Source: BMC Med Educ. 2010 Jun 23;10:48. doi: 10.1186/1472-6920-10-48 (PMC2909973; doi:10.1186/1472-6920-10-48)
Supplement: Additional File 3 — Questionnaire used for survey of respondents. pdf of questionnaire used in survey. [file 1472-6920-10-48-S3.PDF]

The lasting impression of the preparation for house officer

1. Page 1

This research project aims to do the following:
1 To ascertain the lasting impact of the Preparation for House Officer course 2007
2 To discern the relative importance of the taught course and the shadowing period
3 To discern which parts were the most and least useful in Foundation Year 1
4 To report the results to the Preparation for House Officer Steering group in order to influence the future shape and content of the course

To these ends, we would appreciate your filling in this questionnaire.
All responses are entirely confidential between you and the research team and no data which might identify any participant will be shared with anyone else party.
This questionnaire should take less than 10 minutes to complete. Please give your immediate reaction. Don't pause for reflection or to ponder.

1. Which placements have you been in so far and in which hospitals?

First placement

Second placement

Third placement

2. The preparation for house officer course taught course prepared me well for my first foundation doctor job

3. I enjoyed the taught course

4. I found the taught course did not prepare me for working as a foundation doctor

5. The length of the taught course should be...

- One week
- Two weeks
- Three weeks or more

6. The taught course gave me better preparation for working than the shadowing did

7. The shadowing part of the course was more useful than the taught part

8. The taught course brought back to mind

- a lot of important knowledge
- some important knowledge
- a little important knowledge
- no important knowledge

9. How much can I remember that is positive about the taught course?

- a lot
- some
- a little
- nothing at all

10. During my first job I drew on the knowledge I gained from the taught course

11. A lot of what we covered in the taught course should have been done in shadowing

12. The taught course repeated

- a lot of the stuff we should have known already
- some of the stuff we should have known already
- a little of the stuff we should have known already
- none of the stuff we should have known already

The lasting impression of the preparation for house officer

2. page 2

Try to remember as best you can what was in the course but please don't ponder your reply. You can write as much as you like in the boxes below.

13. Which part/s of the taught course do you think were most relevant to your starting work as a Foundation doctor?
Can you explain your response?

14. Which part/s of the shadowing were most relevant to your starting work as a Foundation doctor?
Can you explain your response?

15. Were there any part/s of the taught course that seemed unimportant at the time but which you now think are important for a new Foundation doctor?
Can you explain your response?

16. Were there any part/s of the taught course that seemed important at the time but which you now think are unimportant for a new Foundation doctor?
Can you explain your response?

17. What should have been left out of the taught course?
Can you explain your response?

18. What else should have been included in the taught course?
Can you explain your response?

19. What should have been left out of the shadowing?
Can you explain your response?

20. What else should have been included in the shadowing?
Can you explain your response?

The lasting impression of the preparation for house officer

3. page 3

Below is a list of the taught sessions in the preparation for house officer course. They are more or less in order of presentation. Please use them to answer the last few questions but don't go back and change your previous responses!

Introduction and how to pass the course
Elective debrief
CVS
Community health
Terminal care
Child Protection
Neurological emergencies
Fluids
Sepsis
Setting up your email
Acute care
Surgical emergencies
How to prescribe
Psychiatric emergencies
Respiratory emergencies
GI emergencies
Pain
How to avoid getting struck off
Medicine and the law
Foundation programme
Self-protection
Surviving the NHS
Time management
Complaints and how to avoid them
Deanery talk
European working time directive
Work-life balance
Doctor as patient
Hospital careers
GP careers
How to get the most out of shadowing

21. Which part/s of the taught course do you think were most relevant to your starting work as a Foundation doctor?

Can you explain your response?

22. Were there any part/s of the taught course that seemed unimportant at the time but which you now think are important for a new Foundation doctor?

Can you explain your response?

The lasting impression of the preparation for house officer

23. Were there any part/s of the taught course that seemed important at the time but which you now think are unimportant for a new Foundation doctor?

Can you explain your response?

24. What should have been left out of the taught course?

Can you explain your response?

25. What else should have been included in the taught course?

Can you explain your response?

Thank you for completing this questionnaire. Once we have collated and analysed the results, we will pass them on to Preparation for House Officer Steering Group.
